# Promoter profiles in plasma CfDNA exhibits a potential utility of predicting the efficacy of neoadjuvant chemotherapy in breast cancer patients

**DOI:** 10.1186/s13058-024-01860-3

**Published:** 2024-07-04

**Authors:** Xu Yang, Qing Liu, Zhiwei Guo, Xuexi Yang, Kun Li, Bowei Han, Min Zhang, Minying Sun, Limin Huang, Gengxi Cai, Yingsong Wu

**Affiliations:** 1 Institute of Antibody Engineering, School of Laboratory Medicine and Biotechnology, Southern Medical University, Guangzhou, China; 2 Department of Pathology, The First People’s Hospital of Foshan, Foshan, China; 3 Department of Primary Public Health, Guangzhou Center for Disease Control and Prevention, Guangzhou, China; 4 Institute of Public Health, Guangzhou Medical University & Guangzhou Center for Disease Control and Prevention, Guangzhou, China; 5 Sun Yat-Sen Memorial Hospital, Sun Yat-Sen University, Guangzhou, China

**Keywords:** Promoter profiles, Plasma cfDNA, Treatment efficacy, Neoadjuvant chemotherapy, Non-invasive

## Abstract

**Background:**

Gene expression profiles in breast tissue biopsies contain information related to chemotherapy efficacy. The promoter profiles in cell-free DNA (cfDNA) carrying gene expression information of the original tissues may be used to predict the response to neoadjuvant chemotherapy in breast cancer as a non-invasive biomarker. In this study, the feasibility of the promoter profiles in plasma cfDNA was evaluated as a novel clinical model for noninvasively predicting the efficacy of neoadjuvant chemotherapy in breast cancer.

**Method:**

First of all, global chromatin (5 Mb windows), sub-compartments and promoter profiles in plasma cfDNA samples from 94 patients with breast cancer before neoadjuvant chemotherapy (pCR = 31 vs. non-pCR = 63) were analyzed, and then classifiers were developed for predicting the efficacy of neoadjuvant chemotherapy in breast cancer. Further, the promoter profile changes in sequential cfDNA samples from 30 patients (pCR = 8 vs. non-pCR = 22) during neoadjuvant chemotherapy were analyzed to explore the potential benefits of cfDNA promoter profile changes as a novel potential biomarker for predicting the treatment efficacy.

**Results:**

The results showed significantly distinct promoter profile in plasma cfDNA of pCR patients compared with non-pCR patients before neoadjuvant chemotherapy. The classifier based on promoter profiles in a Random Forest model produced the largest area under the curve of 0.980 (95% CI: 0.978–0.983). After neoadjuvant chemotherapy, 332 genes with significantly differential promoter profile changes in sequential cfDNA samples of pCR patients was observed, compared with non-pCR patients, and their functions were closely related to treatment response.

**Conclusion:**

These results suggest that promoter profiles in plasma cfDNA may be a powerful, non-invasive tool for predicting the efficacy of neoadjuvant chemotherapy breast cancer patients before treatment, and the on-treatment cfDNA promoter profiles have potential benefits for predicting the treatment efficacy.

**Supplementary Information:**

The online version contains supplementary material available at 10.1186/s13058-024-01860-3.

## Background

Neoadjuvant chemotherapy, an important part of the standard treatment, has been used more frequently in the treatment of breast cancer. It is the conventional therapy for patients with locally advanced breast cancer and it aims to shrink tumors to enable surgery and conserve the breast [1]. The tumor response to neoadjuvant chemotherapy is a strong predictive factor of patient outcome and prognosis and mainly assessed according to Response Evaluation Criteria in Solid Tumor version 1.1 (RECIST v1.1) [2]. A pCR (pathological complete response) which is defined as 0% viable tumor cells in the residual tumor after neoadjuvant chemotherapy represents a surrogate marker endpoint for a prediction of good prognosis [3]. Achieving pCR is therefore one of the main objectives of neoadjuvant chemotherapy. However, only a minoring of patients could obtain pCR. There are still many patients with non-pCR [4, 5], and a small proportion of them have no response to neoadjuvant chemotherapy, and some else even develop tumor progression [6, 7]. In addition, the risk of tumor progression and distant metastasis increased in non-pCR patients [8]. Therefore, it is very important to evaluate the response in the pre-treatment or early treatment stage.

Conventional methods for assessing pCR after neoadjuvant chemotherapy mainly consist of magnetic resonance imaging (MRI), ultrasound (US), mammography, and positron emission tomography/computed tomography (PET/CT). However, these imaging methods are limited in accurately assessing the early treatment response, and postoperative pathological examination has a time lag in response to therapeutic effects, which is not conducive to the timely adjustment of treatment strategies [9]. The gold standard for response is the examination of surgically resected specimens. Some reports have attempted to characterize molecular predicting biomarkers at pre-treatment and early treatment of neoadjuvant chemotherapy using tissue specimens [10, 11]. However, the pre- and post-treatment puncture tissue specimens obtained would traumatize the patients. Furthermore, the tissue specimens from the puncture were scarce and difficult to represent the entire genomic landscape of breast tumors. The response monitoring through sequential samples is even more difficult to achieve.

Liquid biopsy may enable sensitive prediction of recurrence and clinical outcomes [12]. CfDNA has been an essential biomarker in many cancer applications, such as early detection and outcome prediction of cancer. A few other reports have suggested that whole genome cfDNA could detect early-stage cancer [13, 14]. CfDNA could provide more comprehensive information because it contains both tumor-derived and non-tumor-derived DNA information [13, 14]. There has been some evidence of the interrelation between non-tumor-derived DNA and cancers, including that some immune-cell apoptosis patterns were found in patients with cancers, and a low lymphocyte-to-monocyte ratio was found to correlate with poor prognosis [15, 16].

Importantly, cfDNA has been considered to carry nucleosomal footprints from the necrotic tumor tissue and apoptotic leukocytes, and the coverage of the promoters could be used to predict gene expression [17–20]. As tumor and immune-cell gene expression are both closely related to the response to cancer therapy and cfDNA has been demonstrated containing tumor-specific and non-tumor-specific open chromatin regions [21], we hypothesized that the promoter profiles in plasma cfDNA could be used for predicting the efficacy of neoadjuvant chemotherapy.

In the present study, we performed an exploratory study to investigate the feasibility of using the promoter profiles in plasma cfDNA for predicting the efficacy of neoadjuvant chemotherapy in breast cancer. We first compared the global chromatin (5 Mb windows), sub-compartments and promoter profiles in plasma cfDNA before treatment between the pCR and non-pCR to neoadjuvant chemotherapy in patients with breast cancer, to identify the potential utility of the promoter profiles for predicting the efficacy of neoadjuvant chemotherapy. Further, we developed classifiers based on the promoter profiles for predicting the efficacy using multiple machine learning models. Finally, we analyzed the promoter profile changes in sequential cfDNA samples during neoadjuvant chemotherapy to explore the potential benefits of on-treatment cfDNA promoter profiles as a novel potential biomarker for predicting the treatment efficacy.

## Methods

### Patients and samples

Tables 1 and 2 provided the clinical characteristics of the patients. A total of 154 retrospective plasma samples from 94 patients with breast cancer including 94 samples before neoadjuvant chemotherapy (T0, pre-treatment) who mainly received adjuvant anthracyclines, cyclophosphamide and paclitaxel regimens, and 60 samples from the above 30 patients at two other time points, post- 3 or 4 cycles of epirubicinneoa/cyclophosphamide (EC) treatment (T1), and subsequent post- 3 or 4 cycles of docetaxel (T) treatment (T2) before surgery, in addition to pre-treatment (T0). The total 154 samples were collected from February 2017 to July 2019 and stored at -80℃ before use. All 94 patients received neoadjuvant chemotherapy followed by surgery. The postsurgical assessment was performed according to the evaluation criteria of the Miller−Payne histological grading system using tissue samples collected during surgery after completion of neoadjuvant chemotherapy [22, 23]. Based on the response to cancer therapy, the participants were divided into two groups: patients with pCR (*n* = 31) and non-pCR (*n* = 63) before neoadjuvant chemotherapy; and patients with pCR (*n* = 8) and non-pCR (*n* = 22) during treatment. Plasma samples were collected from patients at the First People’s Hospital of Foshan, Guangdong, China. Ethical approval was obtained from the Ethics Committee of the First People’s Hospital of Foshan (ethical review number: L[2021] no.5). All the participants provided written informed consent.

**Table 1:** Clinical characteristics of 94 breast cancer patients

	All Patients	pCR	non-pCR	*P*-value
	(*n* = 94)	(*n* = 31)	(*n* = 63)	
Age years, median (IQR)	47 (11)	46 (11)	48 (11)	0.954 ^*^
Histological grade				0.405 ^*^
< 3	73 (77.66%)	23 (74.19%)	50 (79.37%)	
= 3	21 (22.34%)	8 (25.81%)	13 (13.83%)	
HER2 status				0.995 ^#^
Positive	43 (45.74%)	18 (58.06%)	25 (39.68%)	
Negative	51 (54.26%)	13 (41.94%)	38 (60.32%)	
ER status				< 0.001 ^*^
Positive	64 (68.09%)	15 (48.39%)	49 (77.78%)	
Negative	30 (31.91%)	16 (51.61%)	14 (22.22%)	
PR status				0.017 ^*^
Positive	71 (75.53%)	20 (64.52%)	51 (80.95%)	
Negative	23 (24.47%)	11 (35.48%)	12 (19.05%)	
Ki67 degree				0.014 ^*^
≤ 30%	48 (51.06%)	11 (35.48%)	37 (58.73%)	
> 30%	46 (48.94%)	20 (64.52%)	26 (41.27%)	
Adjuvant anthracyclines (E/D), cyclophosphamide (C), paclitaxel (T) regimens				0.365 ^#^
EC-T	62 (65.96%)	18 (58.06%)	44 (69.84%)	
DC-T	15 (15.96%)	6 (19.35%)	9 (14.29%)	
T-EC	8 (8.51%)	4 (12.90%)	4 (6.35%)	
EC	3 (3.19%)	0 (0%)	3 (4.76%)	
TC	1 (1.06%)	0 (0%)	1 (1.59%)	
T	5 (5.32%)	3 (9.68%)	2 (3.17%)	
Adjuvant mabs				0.002 ^#^
Trastuzumab	35 (37.23%)	16 (51.61%)	19 (30.16%)	
Trastuzumab + Patuzumab	3 (3.19%)	3 (9.68%)	0 (0%)	
No	56 (59.57%)	12 (38.71%)	44 (69.84%)	
Adjuvant carboplatin				0.989 ^#^
Yes	3 (3.19%)	1 (3.23%)	2 (3.17%)	
No	91 (96.81%)	30 (96.77%)	61 (96.83%)	
Incomplete Prognostic information (< 3 years outcome)	5	2	3	
Relapse (≥ 3 years outcome)				0.546 ^#^
No	82 (92.13%)	26 (89.66%)	56 (93.33%)	
Yes	7 (7.87%)	3 (10.34%)	4 (6.67.%)	
Relapse-free survival Months, median (IQR)	55 (16)	52 (16)	56 (15)	0.563 ^*^
Disease (≥ 3 years outcome)				0.212 ^#^
No	79 (88.76%)	24 (82.76%)	55 (91.67%)	
Yes	10 (11.24%)	5 (17.24%)	5 (8.33%)	
Survival status (≥ 3 years outcome)				0.484 ^#^
Alive	88 (98.88%)	29 (100%)	59 (98.33%)	
Dead	1 (1.126%)	0 (0%)	1 (1.67%)	

Abbreviations: ^*^ T test (two-tailed); ^#^ Pearson’s chi-squared test; IQR: interquartile range; ER, estrogen receptor; PR, progesterone; HER2, human epidermalgrowth factor receptor-2; EC-T: four cycles of epirubicin (E)/cyclophoshamide (C) followed by four cycles of docetaxel (T); DC-T: four cycles of liposome doxorubicin (D)/cyclophoshamide (C) followed by four cycles of docetaxel (T); EC: four cycles of epirubicin (E)/cyclophoshamide (C); TC: six cycles of docetaxel (T)/cyclophoshamide (C); T: six cycles of docetaxel (T); pCR, pathological complete response; non-pCR, non-pathological complete response

**Table 2:** Clinical characteristics of 30 breast cancer patients undergoing EC-T chemotherapy

	All Patients	pCR	non-pCR	*P*-value
	(*n* = 30)	(*n* = 8)	(*n* = 22)	
Age years, median (IQR)	50 (16)	47 (14)	51 (16)	0.635 ^*^
Histological grade				1.000 ^*^
< 3	22 (73.33%)	6 (75.00%)	16 (72.73%)	
= 3	8 (26.67%)	2 (25.00%)	6 (27.27%)	
HER2 status				1.000 ^#^
Positive	10 (33.33%)	2 (25.00%)	8 (36.36%)	
Negative	20 (66.67%)	6 (75.00%)	14 (63.64%)	
ER status				0.235 ^*^
Positive	21 (70.00%)	4 (50.00%)	17 (77.27%)	
Negative	9 (30.00%)	4 (50.00%)	5 (22.73%)	
PR status				0.673 ^*^
Positive	22 (73.33%)	4 (50.00%)	8 (36.36%)	
Negative	8 (26.67%)	4 (50.00%)	14 (63.64%)	
Ki67 degree				0.122 ^*^
≤ 30%	17 (56.67%)	5 (62.50%))	12 (54.55%)	
> 30%	13 (43.33%)	3 (37.50%)	10 (45.45%)	
Adjuvant mabs				0.232 ^#^
Trastuzumab	6 (20.00%)	2 (25.00%)	4 (18.18%)	
No	24 (80.00%)	6 (75.00%)	18 (81.82%)	

Abbreviations: ^*^ Wilcoxon rank-sum test; ^#^ Pearson’s chi-squared test; IQR: interquartile range; ER, estrogen receptor; PR, progesterone; HER2, human epidermal growth factor receptor-2; EC-T: four cycles of epirubicin (E)/cyclophoshamide (C) followed by four cycles of docetaxel (T); pCR, pathological complete response; non-pCR, non-pathological complete response

### Sample preparation and sequencing of cfDNA

500 µL plasma was obtained from 1 mL peripheral blood in EDTA tubes through two-step centrifugation. The centrifugal parameters were 10 min at 1,600 g, followed by 10 min at 16,000 g, both at 4℃. The plasma was stored at -80℃ before use. 1–5 ng cfDNA was extracted from the entire 500 µL plasma using a QIAamp DNA Blood Midi Kit (Qiagen) per sample and used for library construction using the Life Sciences Ion Xpress Plus Fragment Library Kit (Life Technologies, USA). The libraries were analyzed using a bioanalyzer (Agilent Technologies, Singapore). Sequencing was performed using the Ion PI Hi-Q OT2 200 Kit and Ion PI Hi-Q Sequencing 200 Kit. A total of 6−10 million reads were generated for each cfDNA sample.

### Global chromatin (5 Mb windows) and sub-compartments analysis

The sequencing reads were aligned to the human reference genome (hg19) using TMAP (Torrent Mapping Alignment Program, TMAP), and the PCR (Polymerase Chain Reaction, PCR) duplicates were removed using the SAMtools rmdup function [24]. To remove the biases in coverage attributable to the GC content of the genome, we used LOWESS (Locally Weighted Scatterplot Smoothing, LOWESS) with a span setting of 0.75 for each sample. The differential global chromatin in between the pCR group (*n* = 31) and the non-pCR group (*n* = 63), were first analyzed using the number of reads mapped to each 5-megabase (Mb) region by adding up the GC-adjusted coverage values of the 100-kb bins. The differential sub-compartments from the Hi-C data of GM12878 [25, 26] in between the two groups were calculated using the number of reads mapped to each 100-kilobase (kb) region by adding the GC-adjusted coverage of the 100-kb bins.

### Promoter profiles in plasma cfDNA analysis

The coverage in the promoter region was defined as coverage of -1,000 bp to + 1,000 bp around the transcription start site (TSS), according to RefSeq of the University of California Santa Cruz (UCSC). The coverage values were analyzed using bedtools (ver. 2.17.0) [27]. Subsequently, the promoter profile was normalized by dividing the coverage of the promoter region by the total number of mapped reads. Finally, promoter profile changes between the pCR and non-pCR groups (pCR = 31 and non-pCR = 63) were analyzed.

### Statistical analysis

Wilcoxon rank sum test (two-sided) was used for the analysis of the changes between pCR and non-pCR group. The approach for distinguishing pCR and non-pCR was obtained based on the differential promoter profiles with *P-*value ≤ 0.05 and fold change ≥ 1.2. Principal component analysis (PCA) was performed on the differential genes. Hierarchical clustering was applied to the coverage in the promoter region, using the average-linkage clustering algorithms in Cluster (ver. 3.0). Heat maps were plotted using the pheatmap package in the R software (version 3.0.1). The volcano map was plotted using ggplot2.

### Classifiers for distinguishing pCR and non-pCR groups

Genes with significantly different TSS coverage were first selected through Boruta algorithm, which were defined as “Confirmed” or “Tentative”, and then the classifiers for distinguishing between the pCR and non-pCR groups were developed using Logistic Regression (LR), Random Forest (RF) and Support Vector Machines respectively (SVM). Five-fold cross-validation was used to randomly divide the samples into training and validation sets and evaluate the performance. In the training set, the normalized read count of each TSS was discretized according to the optimal cut-off point before the approach. The optimal cut-off point for each promoter was defined as the maximum value of (sensitivity + specificity)/2 in the training sets. Receiver operating characteristic (ROC) curve analysis was performed to calculate the area under the curve (AUC) of the validation set, using the pROC (version 1.16.2) R package (version 3.5.1). The entire process is repeated 100 times. The classifiers basd on gloabl chromatin (5 Mb windows) and sub-compartments were performed in the same way.

### Functional annotation and enrichment

To explore the function of the corresponding genes of differential TSSs, Gene Ontology (GO), Kyoto Encyclopedia of Genes and Genomes (KEGG) pathway and Gene Set Enrichment Analysis (GSEA) were performed using the R package, clusterProfler (Version 4.2.0) [28]. GO terms and KEGG pathways were obtained from the QuickGo [29] and KEGG [30] websites respectively. GSEA was conducted based on each gene to identify significantly distinct pathways from the GO and KEGG database respectively between pCR and non-pCR groups.

### Survival analysis

Survival analysis was performed at different time points using the Kaplan-Meier test and Cox proportional-hazards model.

## Results

### Differential global chromatin (5 mb windows) and sub-compartments in between pCR and non-pCR group

**Fig. 1:**
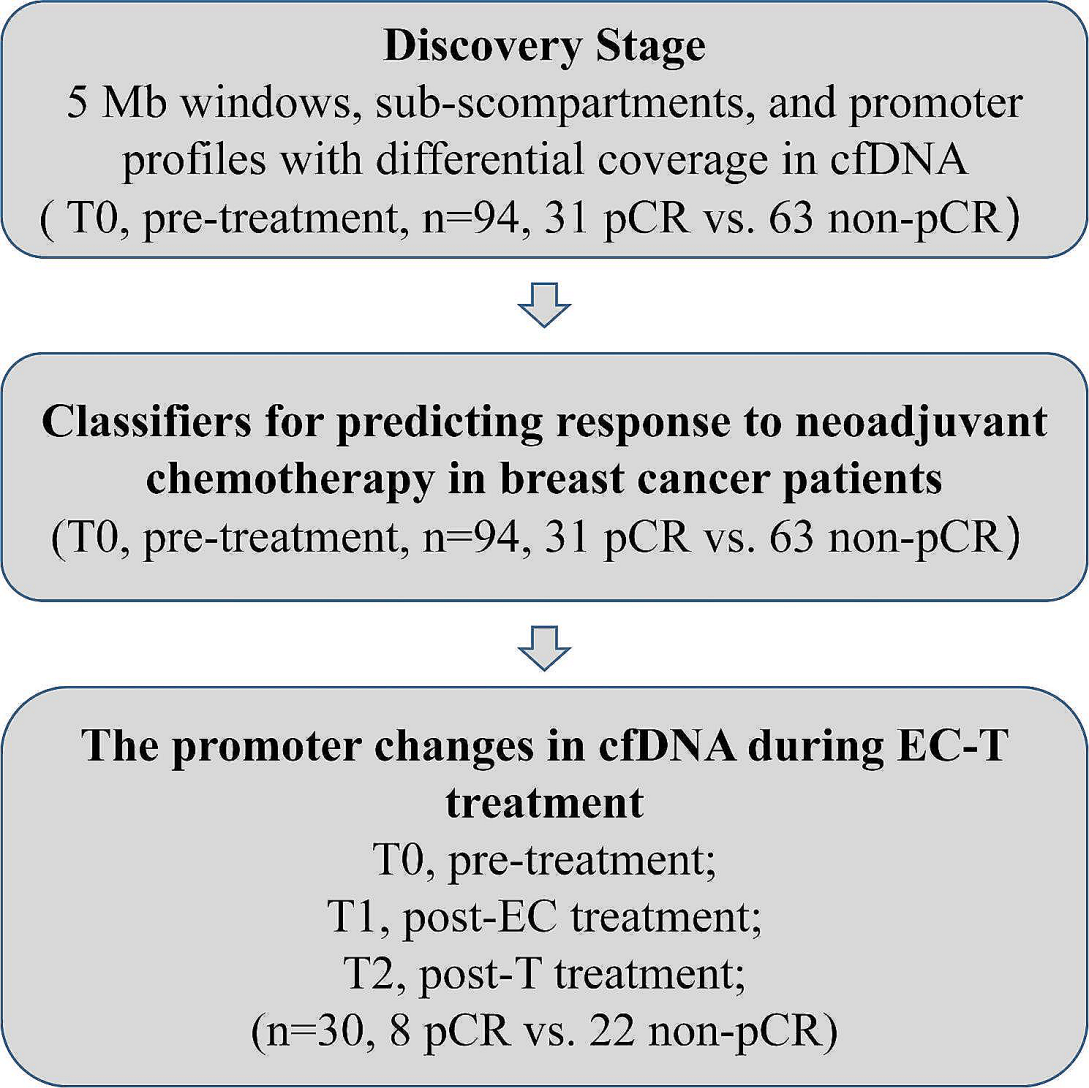
Study design. Our study mainly consisted of three stages, including discovery, validation by developing classifiers and promoter changes analysis in cfDNA during EC-T treatment. In the discovery stage, the genes with differential coverage in cfDNA of between pCR and non-pCR patients were identified. In the validation stage, different classifiers were developed by using the differential features. In the last stage, differential promoter profile changes due to EC-T treatment in cfDNA of between pCR and non-pCR patients were analyzed. cfDNA, cell-free DNA; EC-T, 3 or 4 cycles of epirubicinneoa/cyclophosphamide (EC) treatment and subsequent 3 or 4 cycles of docetaxel treatment before surgery; pCR, pathological complete response; non-pCR, non-pathological complete response

**Fig. 2:**
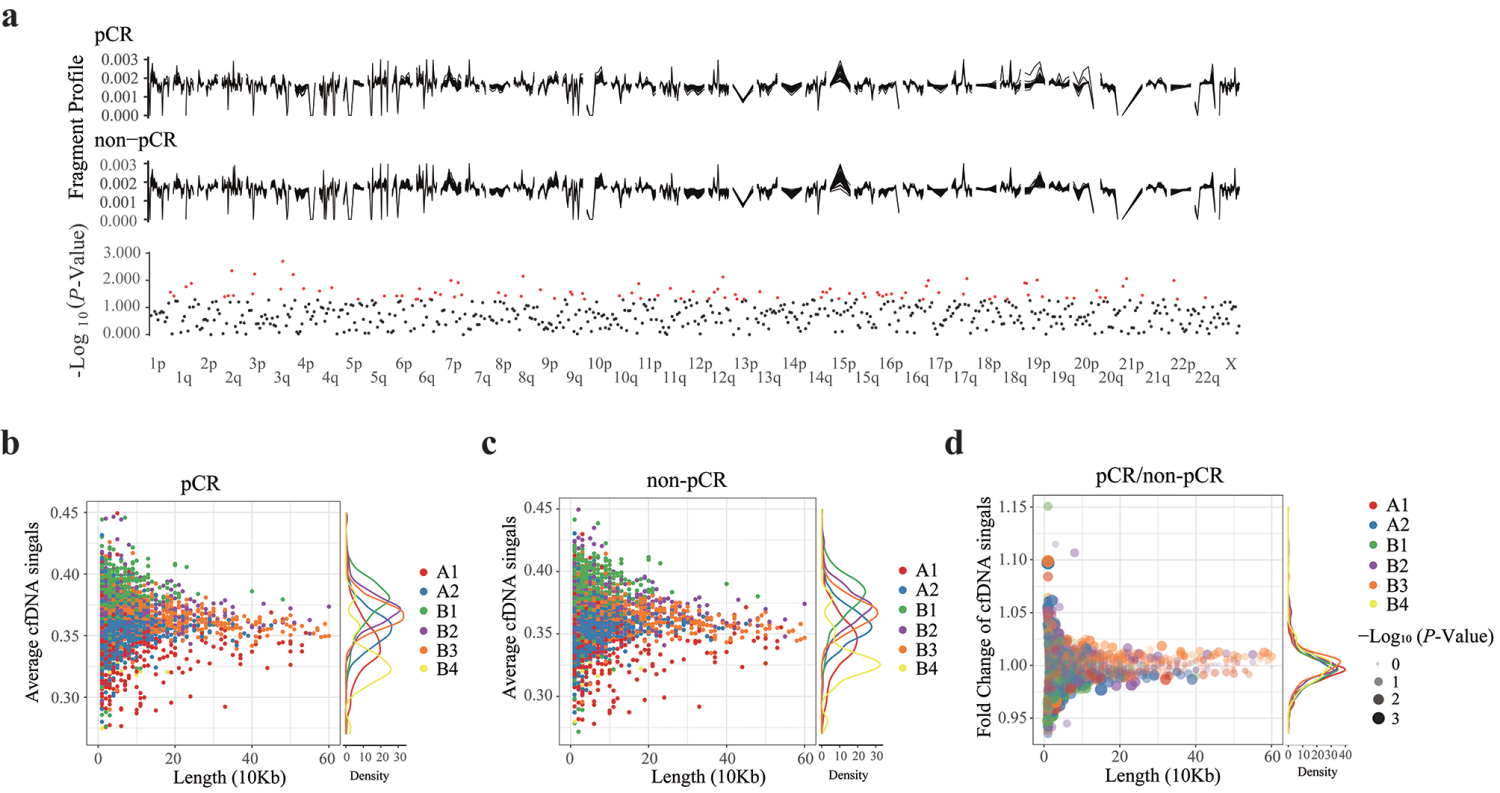
Differential global chromatin (5 Mb windows) and sub-compartments in cfDNA of between pCR and non-pCR patients. **a** Genome-wide fragmentation profiles shown in 5 Mb bins in cfDNA of pCR and non-pCR patients. **b** Sub-compartments in cfDNA of pCR patients. **c**. Sub-compartments annotated in cfDNA of non-pCR patients. **d** The fold change and *P*-value of sub-compartments in cfDNA of pCR versus non-pCR patients. Sub-compartments of the human genome were annotated by the Hi-C data of GM12878. A1 and A2 regions are enriched regions. B1 consists of facultative heterochromatic regions. B2 is enriched at the nuclear lamina and NADs. B3 is also enriched at the nuclear lamina but not at NADs. cfDNA, cell-free DNA; pCR, pathological complete response; non-pCR, non-pathological complete response; cfDNA, cell-free DNA; NADs, nucleolus-associated domains

The workflow of our study mainly consisted of three stages, including discovery, validation by developing classifiers and promoter changes analysis in cfDNA during EC-T treatment (Fig. 1). According to their response to cancer therapy, patients with breast cancer were divided into two groups: pCR and non-pCR groups. As previous studies reported, global chromatin changes occur in different types of cancer [13], and thus there may be global nucleosomal differences between patients with different responses to neoadjuvant therapy. In the discovery, we first compared differential global chromatin of cfDNA in between the pCR group and non-pCR group to neoadjuvant chemotherapy, and we found that there were 98 distinct genomic fragments (*P* ≤ 0.05) with increases and decreases in 5 M windows between the pCR group and non-pCR group, distributed across all the autosomes but chromosome 21 and chromosome X by Wilcoxon rank sum test, with the fold change from 0.95 to 1.06 (Fig. 2a; Additional file 1: Table S1). We then compared the differences in the sub-compartments between the two groups. According to the Hi-C data of GM12878, sub-compartments A1 and A2 consisted of gene-enriched euchromatic regions, B1, B2, and B3 mainly consisted of facultative heterochromatic regions, and B4 was merely present on chromosome 19 [25]. We observed 246 distinct sub-compartments with increased and decreased signals between the pCR group and non-pCR group in all sub-compartment regions (Fig. 2b, c and d), with the fold change from 0.95 to 1.06 (Additional file 1: Table S2).

### Differential promoter profiles in between pCR and non-pCR group

**Fig. 3:**
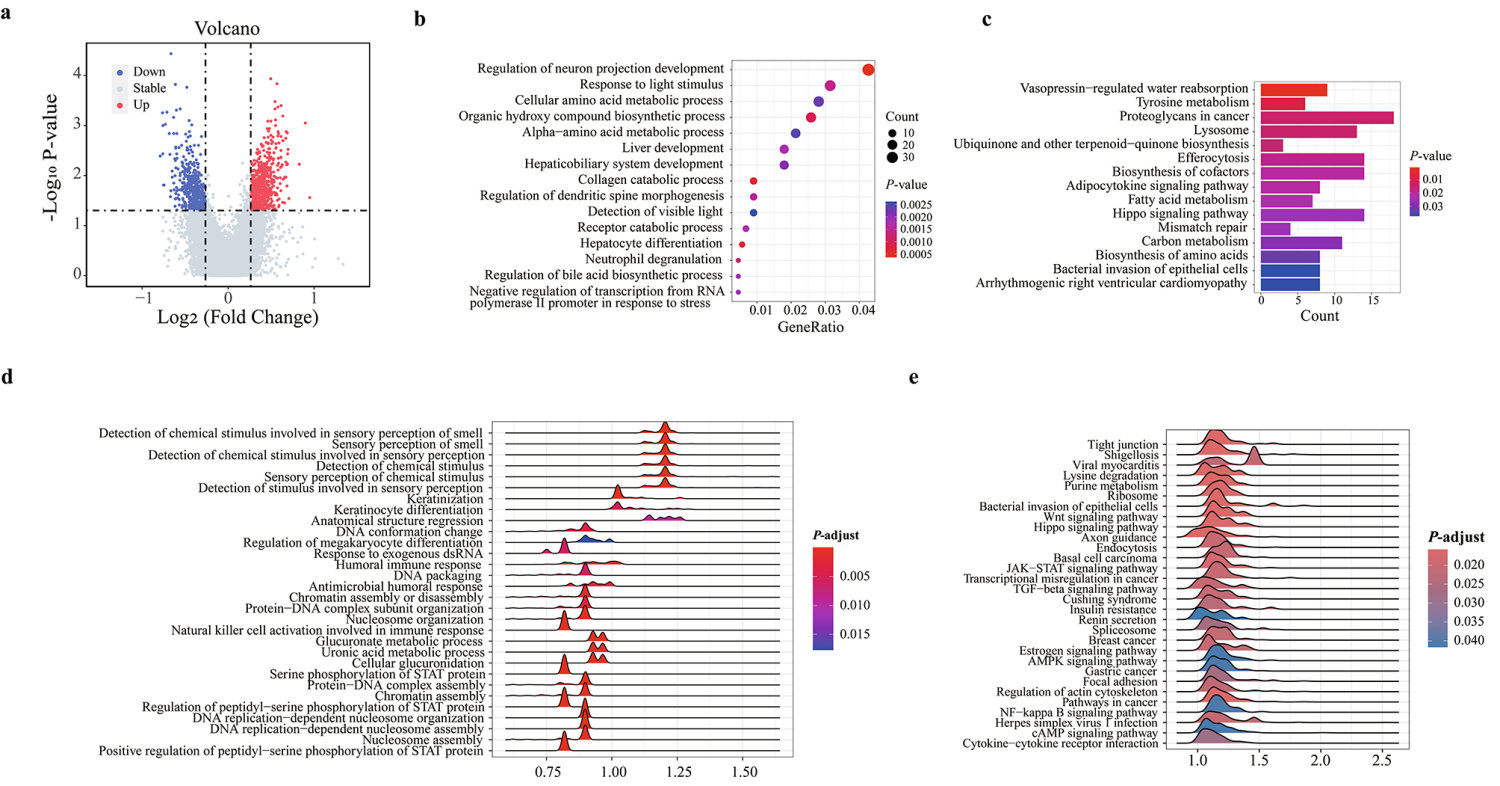
Differential promoter profiles in cfDNA of between pCR and non-pCR patients. **a** Volcano plots of differential promoter profiles (*P*-value ≤ 0.05 and fold change ≥ 1.2). **b** GO enrichment analysis of the differential promoter profiles. **c** KEGG pathway analysis of the differential promoter profiles. **d** GSEA analysis of differential pathways from GO database. **e** GSEA analysis of differential pathways from KEGG database. cfDNA, cell-free DNA; pCR, pathological complete response; non-pCR, non-pathological complete response; GO, Gene Ontology; KEGG, Kyoto Encyclopedia of Genes and Genomes; GSEA, Gene Set Enrichment Analysis

The promoter profiles in the plasma cfDNA are related to the gene expression status [17–20]. CfDNA has been demonstrated to contain the open chromatin regions of tumor-specific and non-tumor-specific promoters [21]. As the molecular expression profiles were different in the tumor tissue between the pCR and non-pCR groups [10, 11], we next compared the promoter profiles in cfDNA between the pCR and non-pCR groups. We analyzed the promoter profiles by calculating the coverage of -1,000 bp to + 1,000 bp around the transcription start site (TSS) across all genes, and identified 1152 TSSs with significantly different coverage between the pCR and non-pCR groups: 675 TSSs with relatively high coverage and 477 TSSs with relatively low coverage in the patients with pCR (Fig. 3a; Additional file 1: Table S3, fold change ≥ 1.2, *P*-value ≤ 0.05, Wilcoxon rank-sum test). PCA analysis revealed that these differential TSSs in the samples from the same group were clustered together, while the samples from different groups were scattered (Additional file 2: Figure S1).

Genes with different coverage aroud TSSs in plasma cfDNA between the pCR and non-pCR groups may play important roles in breast cancer therapy. Gene Ontology (GO) and Kyoto Encyclopedia of Genes and Genomes (KEGG) pathway enrichment analysis revealed that significant genes are mostly associated with metabolic and biosynthetic processes, and some are related to cancer therapy, such as Proteoglycans in cancer and Hippo signaling pathway (Fig. 3b and c; Additional file 1: Table S4; Additional file 1: Table S5). GSEA analysis showed that the differential pathways enriched between pCR and non-pCR groups were the response to chemical stimulus (Fig. 3d, Additional file 1: Table S6), and multiple signaling pathways associated to cancer (Fig. 3e, Additional file 1: Table S7). According to the current literature [17, 31], cfDNA derived mainly from peripheral blood and tumor tissue, and it could reflect the expression status of its original tissues. Thus, the differential promoter profiles may be associated with response to neoadjuvant chemotherapy.

### Classifiers for predicting response to neoadjuvant chemotherapy

**Fig. 4:**
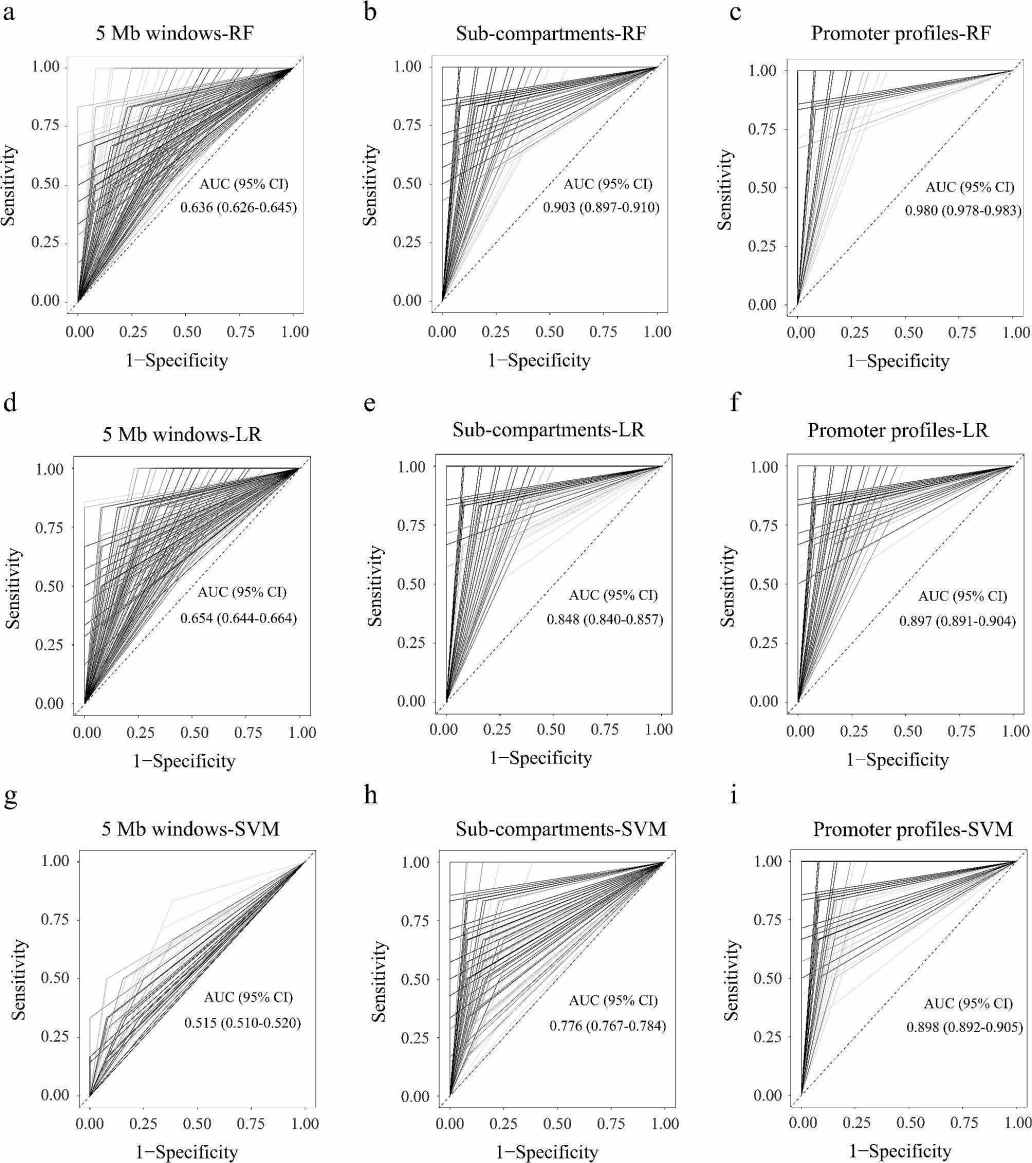
Receiver operating characteristic (ROC) curves of classifiers for distinguishing pCR and non-pCR patients. **a** The classifier based on global chromatin (5 Mb windows) in Random Forest. **b** The classifier based on sub-compartments in Random Forest. **c** The classifier based on promoter profiles in Random Forest. **d** The classifier based on global chromatin (5 Mb windows) in Logistic Regression. **e** The classifier based on sub-compartments in Logistic Regression. **f** The classifier based on promoter profiles in Logistic Regression. **g** The classifier based on global chromatin (5 Mb windows) in Support Vector Machines. **h** The classifier based on sub-compartments in Support Vector Machines. **i** The classifier based on promoter profiles in Support Vector Machines. pCR, pathological complete response; non-pCR, non-pathological complete response; RF: Random Forest; LR: Logistic Regression; SVM, Support Vector Machines

To further evaluate the potential promoter profiles for predicting response to neoadjuvant chemotherapy, we used the differential global chromatin (5 Mb windows), sub-compartments and promoter profiles in plasma cfDNA samples in the discovery stage and developed three classifiers to predict pCR. Five-fold cross-validation was used one hundred times to randomly divide 94 patients into training and validation sets and evaluate the performance. ROC analysis was used to evaluate the area under curve (AUC), accuracy, sensitivity and specificity. Among all combinations, the classifiers based on the promoter profiles of 30 genes had a higher performance, compared with global chromatin (5 Mb windows) and sub-compartments in each LR, RF and SVM model (Fig. 4a and g; Table 3). Across all cohorts, the classifier based on the promoter profiles had a highest AUC value (AUC = 0.980 (95% CI, 0.978–0.983) in Random Forest model, with an accuracy of 0.953 (95% CI, 0.948–0.957), a specificity of 0.943 (95% CI, 0.937–0.950) and a sensitivity of 0.973 (95% CI, 0.968–0.978)) (Fig. 4c; Table 3). The regions used in the classifiers were displayed in Additional file 1: Table S8.

**Table 3:** The features of the classifiers based on machine leaning models

Features	Accuracy (95% CI)	Specifity (95% CI)	Sensitivity (95% CI)
5 Mb windows-RF	0.712 (0.703–0.720)	0.753 (0.736–0.771)	0.642 (0.625–0.659)
Sub-compartments-RF	0.887 (0.880–0.893)	0.901 (0.891–0.911)	0.858 (0.846–0.870)
Promoter profiles-RF	0.953 (0.948–0.957)	0.943 (0.937–0.950)	0.973 (0.968–0.978)
5 Mb windows-LR	0.716 (0.707–0.724)	0.728 (0.711–0.746)	0.692 (0.675–0.709)
Sub-compartments-LR	0.833 (0.826–0.840)	0.822 (0.810–0.833)	0.856 (0.843–0.869)
Promoter profiles-LR	0.880 (0.874–0.887)	0.870 (0.861–0.880)	0.901 (0.891–0.911)
5 Mb windows-SVM	0.538 (0.527–0.550)	0.582 (0.550–0.614)	0.449 (0.418–0.480)
Sub-compartments-SVM	0.829 (0.823–0.836)	0.936 (0.929–0.943)	0.614 (0.598–0.630)
Promoter profiles-SVM	0.918 (0.913–0.923)	0.956 (0.950–0.961)	0.841 (0.829–0.854)

Abbreviations: RF: Random Forest; LR: Logistic Regression; SVM, Support Vector Machines

### The promoters in plasma cfDNA associated with long-term outcome

**Fig. 5:**
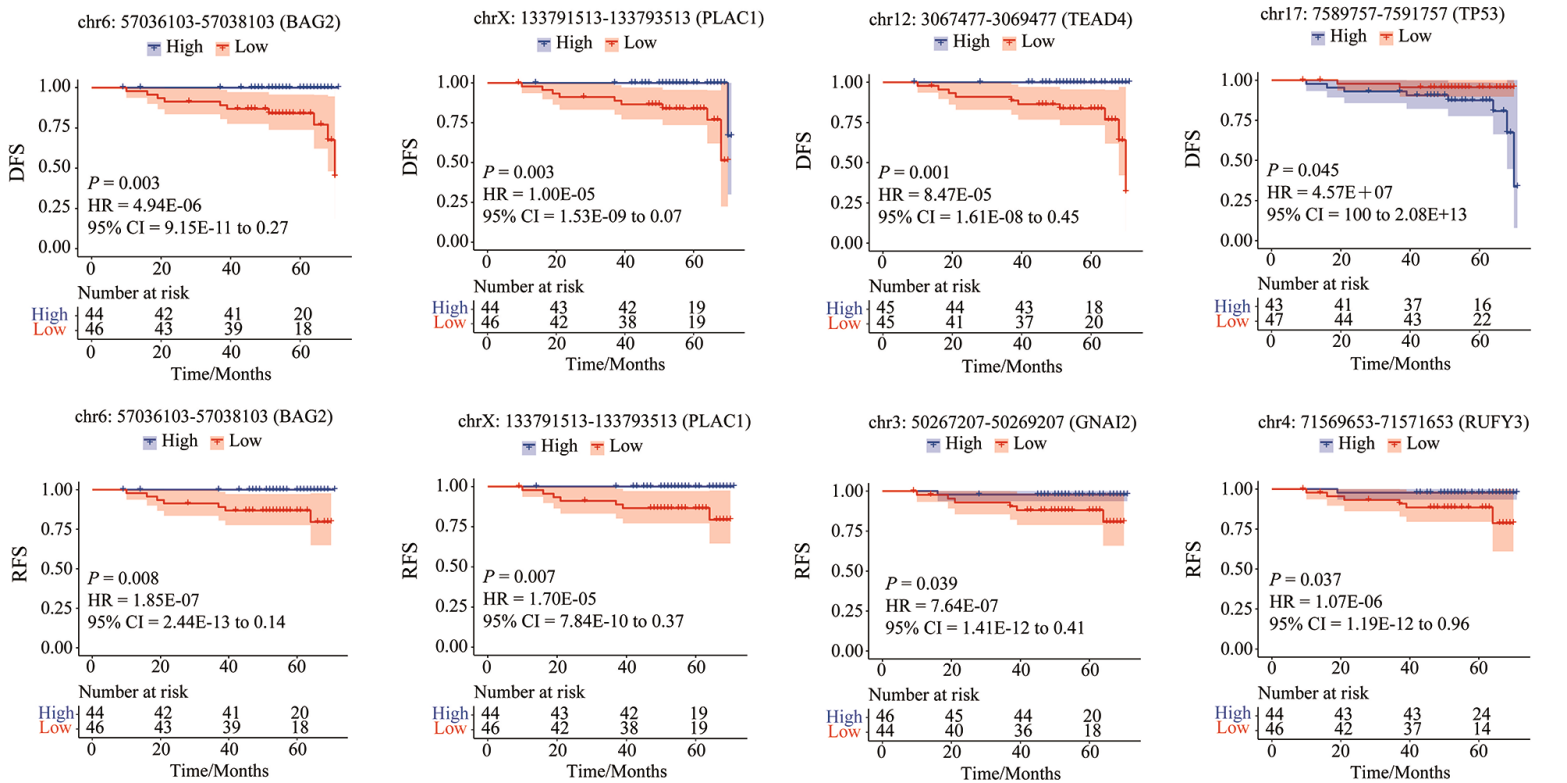
Disease-free survival (DFS) and relapse-free survival (RFS) for BAG2, TRIM35, TEAD4, TP53, GNAI2 and RUFY3. HR, hazard ratio; pCR, pathological complete response; non-pCR, non-pathological complete response

The follow-up data up to 60 months for the patients was collected. In view of this, we performed Kaplan-Meier analysis and observed 370 TSSs associated with relapse-free survival (P_KM_ ≤0.05, P_HR_ ≤0.05) (Additional file 1: Table S9) and 399 TSSs associated with disease-free survival (P_KM_ ≤0.05, P_COX_ ≤0.05) (Additional file 1: Table S10). However, the total number of relapses and diseases was limited and not significantly associated with pCR (RFS, *P* = 0.563; DFS, *P* = 0.212), we focused only several genes, which were related to prognosis in the previous reports [32–37]. The results showed that the high coverage of promoters in BAG2 and TRIM35 gene was significantly associated with both RFS and DFS. The high coverage in TEAD4 and the low coverage in TP53, was significantly associated with RFS. The high coverage in GNAI2 and RUFY3 gene was significantly associated with DFS (Fig. 5). These genes were often highly expressed in many tumors, and associated with prognosis. For example, TP53 as a tumor suppressor, was significantly associated with good prognosis [32]. Our approach showed that the DFS in the group with high coverage of promoter in TP53 in plasma cfDNA was significantly shorter than that with low coverage. This suggest that TP53 may be down-expressed in tumor tissues.

### Differential changes of promoter profiles in plasma cfDNA of patients during EC-T neoadjuvant chemotherapy in between pCR and non-pCR group

**Fig. 6:**
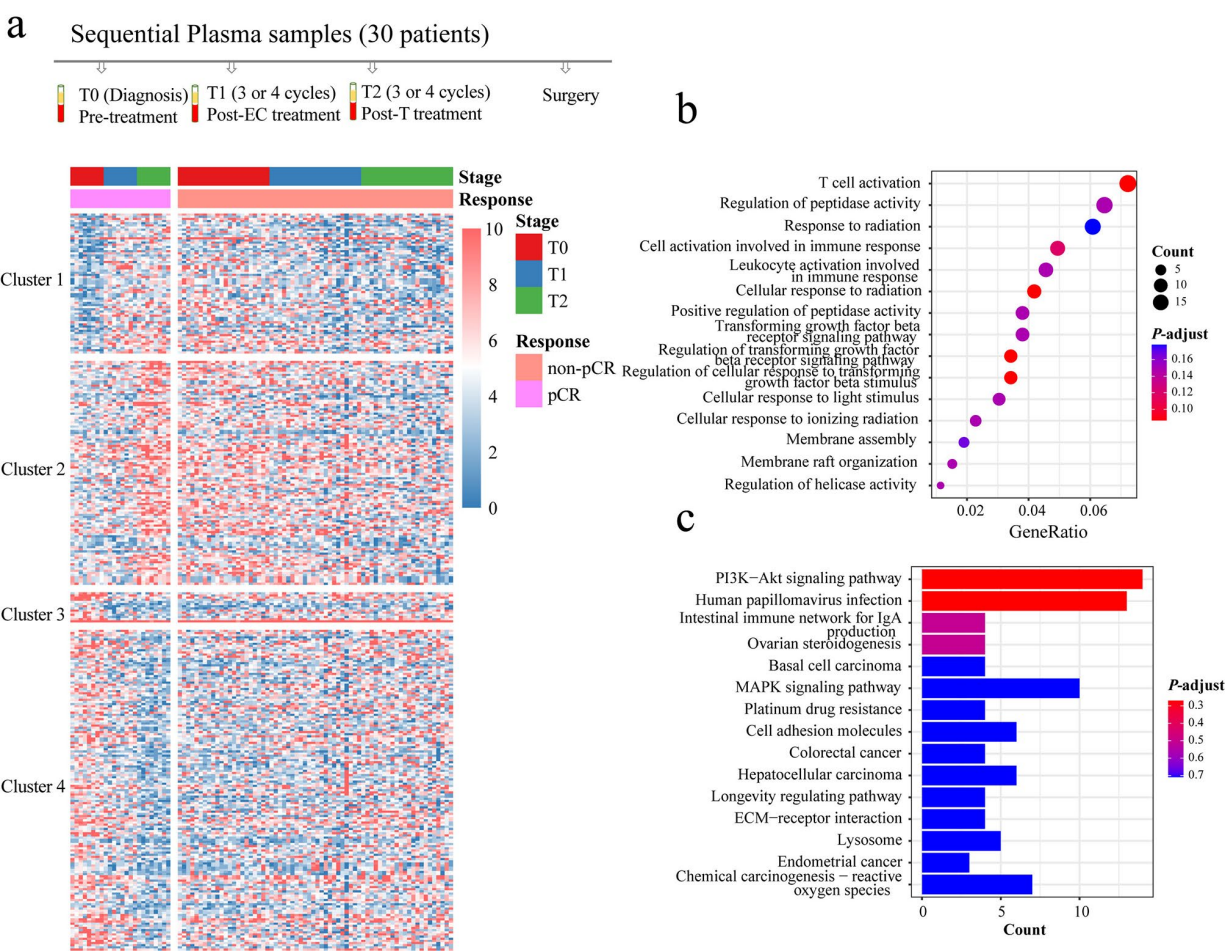
Differential changes of promoter profiles in cfDNA of between pCR and non-pCR patients during EC-T neoadjuvant chemotherapy. **a** Heat map of the z-scores of cfDNA promoters with differential read coverage changes. **b** GO enrichment analysis of the differential promoter changes. **c** KEGG pathway analysis of the differential promoter changes. cfDNA, cell-free DNA; pCR, pathological complete response; non-pCR, non-pathological complete response; GO, Gene Ontology; KEGG, Kyoto Encyclopedia of Genes and Genomes

To explore whether the changes of cfDNA promoter profiles have the potential benefits of predicting the treatment efficacy, we analyzed 90 matched sequential cfDNA samples from 30 patients (pCR = 8 vs. non-pCR = 22) during neoadjuvant chemotherapy at three time points: pre-treatment (T0), post- 3 or 4 cycles of epirubicinneoa/cyclophosphamide (EC) treatment (T1), and subsequent post- 3 or 4 cycles of docetaxel (T) treatment (T2) before surgery. We compared the cfDNA promoter profile changes in between pCR group and non-pCR group, due to EC treatment and T treatment respectively. In total, 65 up-regulated TSSs and 14 down-regulated TSSs during EC treatment, and 104 up-regulated and 149 down-regulated TSSs during T treatment in pCR patients were observed (*P* ≤ 0.05, fold change > 1.2) (Fig. 6a and Additional file 1: Table S11).

GO enrichment analysis based on these total 332 differential changes revealed they are mostly associated with cell response and immune response to treatment, such as T cell activation, response to radiation, cell activation involved in immune response, leukocyte activation involve in immune response, cellular response to radiation, cellular response to light stimulus and cellular response to ionizing radiation. Some are related to cell growth, such as regulation of peptidase activity and membrane assembly (Fig. 6b; Additional file 1: Table S12). KEGG pathway revealed that the changes were enriched in multiple pathways, such as PI3K-Akt signaling pathway, and MAPK signaling pathway (Fig. 6c; Additional file 1: Table S13). Previous studies [38, 39] have shown these pathways were closely related to the patient’s response to cancer therapy. These results may indicate that promoter profile changes during treatment may be useful for predicting the effectiveness of cancer therapy.

**Fig. 7:**
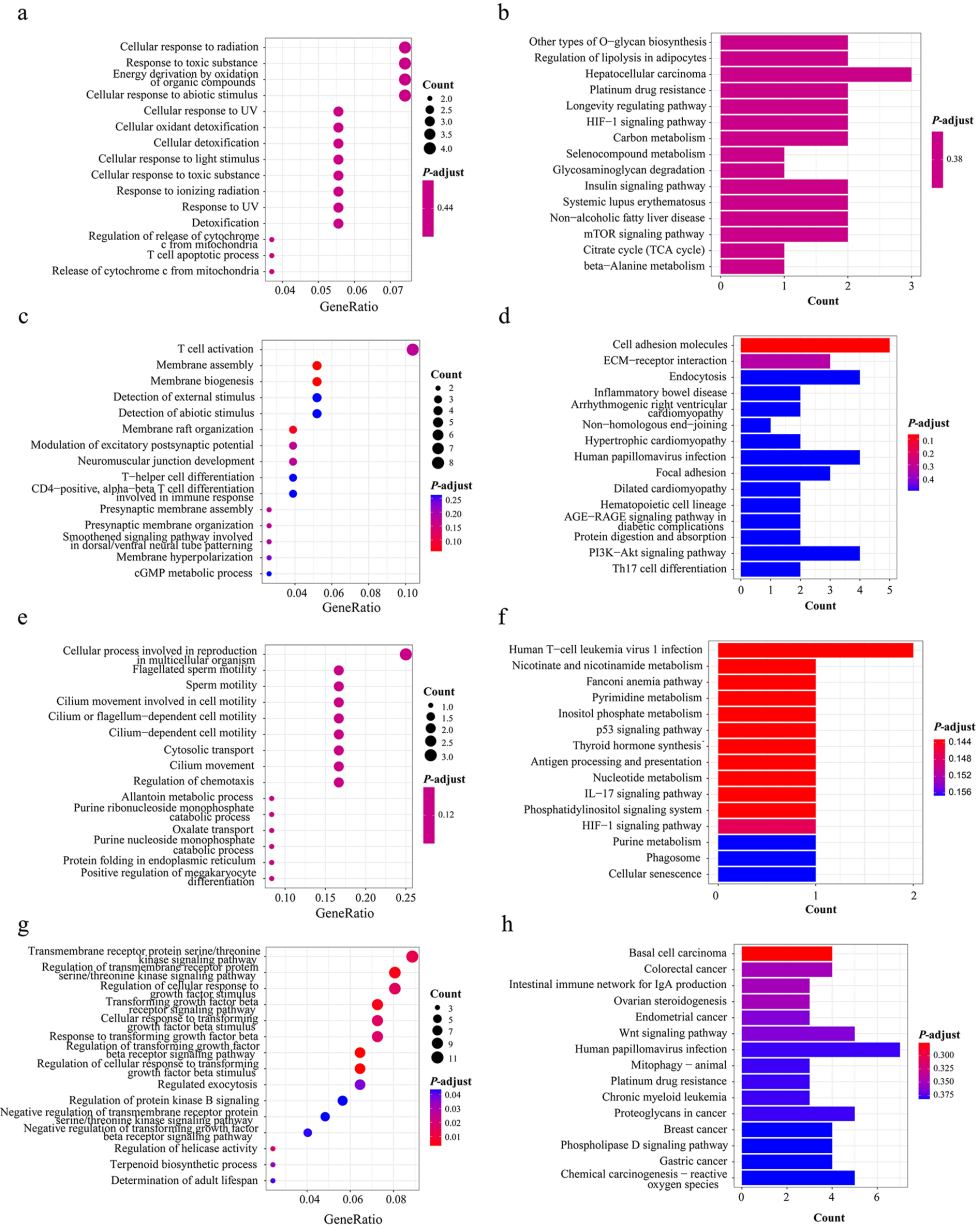
GO enrichment and KEGG pathway analysis for each cluster. **a** GO enrichment analysis for cluster 1; **b** KEGG pathway analysis for cluster 1; **c** GO enrichment analysis for cluster 2; **d** KEGG pathway analysis for cluster 2; **e** GO enrichment analysis for cluster 3; **f** KEGG pathway analysis for cluster 3; **g** GO enrichment analysis for cluster 4; **h** KEGG pathway analysis for cluster 4. Cluster 1, the coverage of promoter profiles was up-regulated due to EC treatment at the first stage in pCR group; cluster 2, the coverage of promoter profiles was up-regulated due to T treatment at the second stage in pCR group; cluster 3, the coverage of promoter profiles was down-regulated due to EC treatment at the first stage in pCR group; cluster 4, the coverage of promoter profiles was down-regulated due to T treatment at the second stage in pCR group; pCR, pathological complete response

We further performed GO and KEGG analysis for each cluster, due to EC treatment at the first stage (Cluster 1 and Cluster 3) and T treatment at the second stage (Cluster 2 and Cluster 4) (Fig. 6a). 65 genes with up-regulated TSSs in Cluster 1 are mostly associated with cell response and metabolism (Fig. 7a and b), and 14 genes with down-regulated TSSs in Cluster 3 are mostly associated with cell motility and metabolism (Fig. 7e and f) after EC treatment. 104 genes with up-regulated TSSs in Cluster 2 are mostly associated with immune response to treatment, such as T cell activation (Fig. 7c and d), and 149 genes with down-regulated TSSs in Cluster 4 are mostly associated with TGF-β signaling pathway (Fig. 7g and h) after T treatment.

## Discussion

Currently, clinical parameters, such as tumor size, estrogen, or HER-2 receptor status, histologic or nuclear grade, and the expression of single molecular markers, show a weak association with the response, limiting their utility in selecting chemotherapy treatment. Multigene molecular expression predictors in tissue samples are more regimen-specific and have been used according to the guidelines for breast cancer published by the National Comprehensive Cancer Network (NCCN) [40] and the American Joint Committee on Cancer [41] in 2019: the Oncotype DX 21-gene assay, Mamma Print 70-gene assay, Endo-Predict 12-gene assay, PAM 50 (Prosigna), and Breast Cancer Index (BCI) tests. However, there are currently few noninvasive diagnostic approaches available for breast cancer response to neoadjuvant chemotherapy predicting. Circulating tumor DNA (ctDNA) generally represents a small fraction of all plasma cell-free DNA (cfDNA), ranging from ≥ 5−10% in late-stage cancer to ≤ 0.01−1.0% in early-stage cancer [42]. In this study, we provide a new non-invasive method for predicting the response to chemotherapy based on expression-specific nucleosomal footprints in plasma cfDNA.

The necrotic tumor tissue and apoptotic leukocytes would generally released their DNA into plasma. Thus nucleosomal footprints of leukocytes cells and tumor cells were reflected in plasma cfDNA. A pCR is currently considered to be the best early outcome after neoadjuvant therapy [43]. Through the analysis of the correlation of nucleosomal footprints in plasma cfDNA and pCR acheiving, we found that there were significant differences in the global chromatin (5 M windows), sub-compartments, and promoter profiles of cfDNA in the pre-treatment stage between pCR and non-pCR patients. A similar finding was observed in patients with colorectal cancer patients treated with neoadjuvant chemoradiotherapy [31]. The genes with differential promoter coverage were enriched in metabolic and biosynthetic processes, and some are related to cancer therapy, such as Proteoglycans in cancer and Hippo signaling pathway (Fig. 3b and d; Additional file 1: Table S4; Additional file 1: Table S5). Previous studies have shown that these pathways are closely associated with the response of patients to cancer therapy. For instance, proteoglycans are attractive pharmacological targets [44]. Hippo signaling pathway might be a therapeutic target [45]. These results may indicate that promoter profiles may be useful for predicting the effectiveness of cancer therapy before treatment.

Further, we used global chromatin (5 M windows), sub-compartments, and promoter profiles in plasma cfDNA and developed classifiers for distinguishing between pCR and non-pCR to neoadjuvant chemotherapy based on the size of 94 patients. The result showed that one classifier based on promoter profiles in plasma cfDNA presented the high performance with the maximum AUC (AUC = 0.897, 95% CI: 0.891–0.904), compared with those based on global chromatin (5 Mb windows) and sub-compartments (Fig. 4). There are 30 genes in the classifier, and these genes are closely associated with the treatment efficacy (Additional file 1: Table S8). We further analyzed several genes related to prognosis, such as BAG2, TRIM35, TEAD4, TP53, GNAI2 and RUFY3. These genes were often highly expressed in many tumors, and associated with prognosis, the results were consistent with previous reports [32–37]. Thus we demonstrated that plasma cfDNA contains information on the efficacy of neoadjuvant chemotherapy before treatment, and the promoter profiles in plasma cfDNA might be an effective tool for predicting the efficacy of neoadjuvant chemotherapy in breast cancer.

We also noted some significantly differential changes of promoter profiles in plasma cfDNA of patients during neoadjuvant chemotherapy in between pCR group and non-pCR group. GO enrichment and KEGG pathway analysis revealed that the related genes are mostly associated with cell response, immune response to treatment, and response to cancer therapy. The genes in tumor tissues and immune cells in the patients who responded differently to treatment would be expressed differently. And further, the promoter profiling in plasma cfDNA could reflect the gene expression in original tumor tissues and immune cells. By further GO enrichment and KEGG pathway analyzing each cluster, cell response pathway to treatment is mainly concentrated in Cluster 1 (Fig. 7a and b), in which the coverage of promoter profiles was up-regulated due to EC treatment at the first stage in pCR group. Often, patients responded strongly at early stage in the whole course of treatment. Immune response immune response mainly concentrated in Cluster 2 (Fig. 7c and d), in which the coverage of promoter profiles was up-regulated due to T treatment at the second stage in pCR group. During the course of treatment, the immune system is subsequently stimulated at a later stage. Thus, these results suggests that promoter profile changes during treatment may be useful for predicting the effectiveness of cancer therapy.

Our study has also some limitations. First, as a limitation of our sample size, we only separated patients into pCR and non-pCR groups. However, patients with non-pCR show different degrees of sensitivity to cancer therapy. Further imaging and pathological evaluation are necessary for surgical management after neoadjuvant chemotherapy. Second, the small sample size limits us to perform the validity of the cohort. The approach for distinguishing between the pCR and non-pCR groups should be validated with more independent cohorts before its clinical application.

## Conclusions

In summary, promoter profiles in plasma cfDNA is a powerful, non-invasive tool for predicting the efficacy of neoadjuvant chemotherapy breast cancer patients before treatment, and the on-treatment cfDNA promoter profiles have potential benefits for predicting the treatment efficacy. Our method based on promoter profiles is promising for assessing the response of patients with breast cancer to therapy before treatment and at early stage during treatment and it is a non-invasive technique that requires only low-coverage DNA sequencing and avoids cancer heterogeneity. Therefore, our method may help prevent the indiscriminate use of drugs, reduce toxicity and side effects, and improve curative effects and quality of life.

### Electronic supplementary material

Below is the link to the electronic supplementary material.

Supplementary Material 1

Supplementary Material 2

## Data Availability

All datasets generated for this study are included in the article/additional material.
