# Supplementary material for: Promoter profiles in plasma CfDNA exhibits a potential utility of predicting the efficacy of neoadjuvant chemotherapy in breast cancer patients
Source: Breast Cancer Res. 2024 Jul 4;26:112. doi: 10.1186/s13058-024-01860-3 (PMC11225256; doi:10.1186/s13058-024-01860-3)
Supplement: Supplementary file 2 — Supplementary Material 2 [file 13058_2024_1860_MOESM2_ESM.pdf]

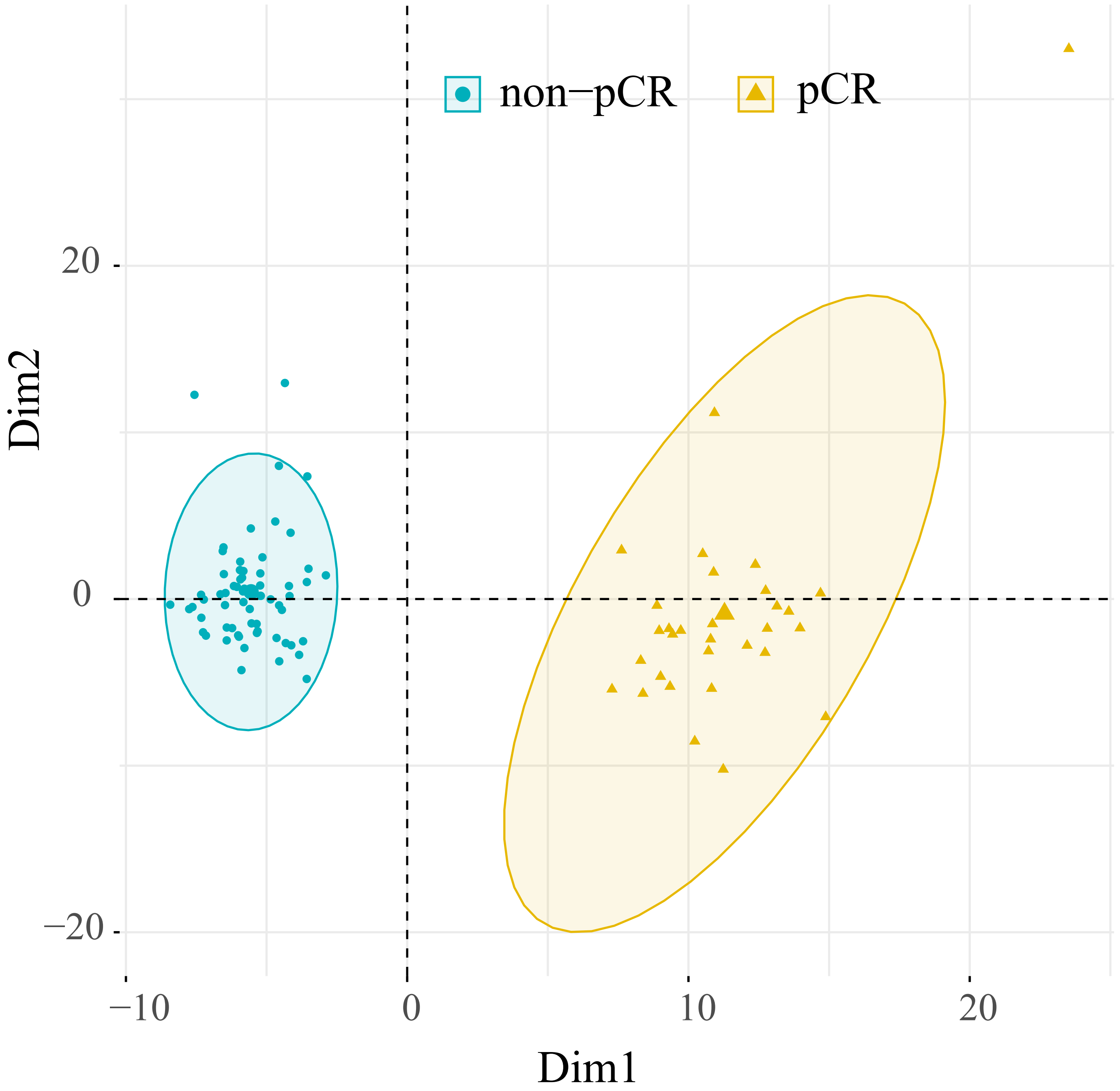

Figure S1. PCA analysis of the differential promoter profiles
pCR, pathological complete response; non-pCR, non-pathological complete response; PCA, principal component analysis.
